# P13 - Vaccination against Haemophilus B as a treatment of recurrent upper respiratory tract infections

**DOI:** 10.1186/2045-7022-4-S1-P68

**Published:** 2014-02-28

**Authors:** Chrys Tsakona

**Affiliations:** 1 Dudley Group of Hospitals NHS Foundation Trust, Dudley, United Kingdom

## 

Haemophilus influenza b (Hib) conjugate vaccines are highly immunogenic in infants, as >95% develop protective antibody levels after 2 or 3 doses; hence invasive Hib disease is now uncommon in vaccinated children. The precise protective level is not clearly established, although a titre of 1 μg/mL is considered to offer long-term protection. Hib vaccination above the age of 5 is not recommended; the majority of older even unvaccinated children seem to be immune to Hib, probably from asymptomatic infections in infancy. The vaccine though is used in any age as a functional assay of the responsiveness of the immune system to capsular polysaccharide antigens, We present 3 children with recurrent upper respiratory tract infections where the test vaccine has also acted in a therapeutic manner.

1) A 6 yr old boy was referred to us for urticaria. His mother mentioned frequent “colds” requiring antibiotics several times a year. His serum Immunoglobulins and routine investigations were normal/negative except for the functional Hib antibody levels which were suboptimal at 0.22 μg/mL rising to >10 μg/mL 4 weeks post-vaccination, when the recurrent infections stopped.

2) Another 6 yr old was referred with eczema and the mother mentioned that he had been investigated inconclusively for unexplained lethargy. He was blowing green mucus from his “constantly blocked nose”; his functional Hib antibody levels were suboptimal at <0.15 rising eventually to 1.7 μg/mL after 3 vaccinations, which stopped the subclinical sinusitis and of course the lethargy.

3) A third child was seen for nut allergy. He missed his follow-up appointments for 18 months because he was constantly having respiratory infections, which caused bronchiectasis. A sputum sample at the time of the diagnosis grew Haemophilus (not typed) and started prophylactic antibiotics. A blood test a month later showed suboptimal levels of functional antibody and subsequently excellent response to the vaccination; the infections stopped, although one could argue this was an effect of the prophylactic antibiotics.

Our relevant adult and paediatric data is currently being studied and processed as this is a new therapeutic aspect.

